# 

**Published:** 1873-03

**Authors:** 


Books, etc., Received.—We have received a number of books, etc., which we had hoped to notice in this number of the Journal, but it has been found impracticable to give them such attention as a critical notice would have required. We beg indulgence from those who have forwarded these publications, with the promise that we shall be better able in the future to give special attention this department. More elaborate and thorough reviews of books received, hereafter, may be expected as a conspicuous feature of the Journal.

